# Acknowledgment to the Reviewers of GMS Zeitschrift für Medizinische Ausbildung 

**DOI:** 10.3205/zma000966

**Published:** 2015-05-13

**Authors:** Martin R. Fischer, Götz Fabry

**Affiliations:** 1 GMS Zeitschrift für Medizinische Ausbildung, Schriftleitung, Erlangen, Deutschland; 2 Klinikum der Universität München, Institut für Didaktik und Ausbildungsforschung in der Medizin, München, Deutschland; 3 GMS Zeitschrift für Medizinische Ausbildung, Stellvertretender Schriftleiter, Erlangen, Deutschland; 4 Albert-Ludwig-Universität Freiburg, Medizinische Fakultät, Abteilung für Medizinische Psychologie und Soziologie, Freiburg, Deutschland

## Acknowledgement

We gratefully acknowledge the valuable contribution of the following peer reviewers, who supported our Journal in 2014. We are looking forward to continuing our collaboration!

## Reviewers in alphabetical order

- Harald Affeldt, Mainz
- Klaus Albegger, Graz
- Matthias Angstwurm, München
- Cadja Bachmann, Hamburg
- Daniel Bauer, München
- Erika Baum, Marburg
- Beatrice Beck Schimmer, Zürich
- Jan Becker, Münster
- Antje Bergmann, Dresden
- Silke Biller, Basel
- Martin Boeker, Freiburg i. Br.
- Klaus Böhme, Freiburg
- Thomas Böker, Heidelberg
- Stefan Bösner, Marburg
- Georg Breuer, Erlangen
- Rainer Büscher, Essen
- Jean-Francois Chenot, Greifswald
- Ulrich Decking, Düsseldorf
- Renate Deinzer, Gießen
- Peter Dieter, Dresden
- Jörg Eberhard, Hannover
- Petra Eggensperger, Heidelberg
- Jan P. Ehlers, Witten
- Götz Fabry, Freiburg
- Pedro M. Faustmann, Bochum
- Martin R. Fischer, München
- Volkhard Fischer, Hannover
- Hendrik Friederichs, Münster
- Franziska Geiser, Bonn
- Orsolya Genzel, München
- Waltraud Georg, Berlin
- Matthäus Grasl, Wien
- Dominik Groß, Aachen
- Christian Gruber, Hannover
- Markus Gulich, Ulm
- Petra Hahn, Freiburg
- Eckhart G. Hahn, Erlangen
- Wolfgang Hampe, Hamburg
- Sigrid Harendza, Hamburg
- Wolf Hautz, Zürich
- Axel Heller, Dresden
- Johanna Christine Hissbach, Hamburg
- Angelika Hofhansl, Wien
- Elisabeth Höfler, Bad Ditzenbach
- Matthias Holzer, München
- Henrike Hölzer, Berlin
- Thorsten Hornung, Bonn
- Bert Huenges, Bochum
- Sören Huwendiek, Bern
- Daniel Ithaler, Graz
- Ernst Jünger, Zürich
- Ingo Just, Hannover
- Andreas Jüttemann, Halle
- Sylvia Kaap-Fröhlich, Zürich
- Martina Kadmon, Oldenburg
- Janine Kahmann, Heidelberg
- Axel Karenberg, Köln
- Gudrun Karsten, Kiel
- Claudia Kiessling, München
- Michael Knipper, Giessen
- Sarah König, Göttingen
- Markus Krautter, Heidelberg
- Carsten Kruschinski, Hannover
- Olaf Kuhnigk, Hamburg
- Sandy Kujumdshiev, Zürich
- Manfred Künzel, Bern
- Claas Lahmann, München
- Martin Lischka, Wien
- Marcus Löwe, Dresden
- Georg Lürs, Hamburg
- Cornelia Mahler, Heidelberg
- Manuela Mandl, Graz
- Simone Manhal, Graz
- Jörg Marienhagen, Regensburg
- Claudia Mews, Hamburg
- Lukas Peter Mileder, Graz
- Ulrike Nachtschatt, Innsbruck
- Marcus Neudert, Dresden
- Christoph Nikendei, Heidelberg
- Zineb Miriam Nouns, Bern
- Udo Obertacke, Mannheim
- Falk R. Ochsendorf, Frankfurt/Main
- Wolfgang Öchsner, Ulm
- Harm Peters, Berlin
- Mona Pfeiffer, München
- Ulrich Pohl, München
- Éva Rásky, Graz
- Tobias Raupach, Göttingen
- Gilbert Reibnegger, Graz
- Anja Rogausch, Bern
- Marco Roos, Erlangen
- Thomas Rotthoff, Düsseldorf
- Stefan Rüttermann, Düsseldorf
- Thorsten Schäfer, Bochum
- Christian Scheffer, Witten
- Petra Scheutzel, Münster
- Sarah Schiekirka, Göttingen
- Kai Schnabel, Bern
- Bettina Schöne-Seifert, Münster
- Janette Schönfeld, Heidelberg
- Hanna Schröder, Aachen
- Christian Schulz, Düsseldorf
- Johannes Schulze, Frankfurt/Main
- Katrin Schüttpelz-Brauns, Mannheim
- Melanie Simon, Aachen
- Dirk Sommerfeldt, Hamburg
- Ulrike Sonntag, Berlin
- Kai Sostmann, Berlin
- Robin Stark, Saarbrücken
- Florian Steger, Halle
- Christoph Stosch, Köln
- Sylvia Stracke, Greifswald
- Juerg Streuli, Zurich
- Anwar Syed Ali, Frankfurt/Main
- Ara Tekian, Chicago
- Sevgi Timbil, Türkei
- Gunther Weitz, Lübeck
- Hans-Jürgen Wenz, Kiel
- Tewes Wischmann, Heidelberg
- Ulrich Woermann-Walthert, Bern
- Michaela Zupanic, Witten

